# Omicron‐specific mRNA vaccine induced cross‐protective immunity against ancestral SARS‐CoV‐2 infection with low neutralizing antibodies

**DOI:** 10.1002/jmv.28370

**Published:** 2022-12-10

**Authors:** Kuan‐Yin Shen, Chung‐Hsiang Yang, Chiung‐Tong Chen, Hui‐Min Ho, Fang‐Feng Chiu, Chiung‐Yi Huang, Hung‐Chun Liao, Chia‐Wei Hsu, Guann‐Yi Yu, Ching‐Len Liao, Hsin‐Wei Chen, Ming‐Hsi Huang, Shih‐Jen Liu

**Affiliations:** ^1^ National Institute of Infectious Diseases and Vaccinology National Health Research Institutes Miaoli Taiwan; ^2^ Institute of Biotechnology and Pharmaceutical Research National Health Research Institutes Miaoli Taiwan; ^3^ Graduate Institute of Biomedical Sciences China Medical University Taichung Taiwan; ^4^ Graduate Institute of Medicine, College of Medicine Kaohsiung Medical University Kaohsiung Taiwan

**Keywords:** COVID‐19, mRNA vaccine, Omicron, SARS‐CoV‐2

## Abstract

The major challenge in COVID‐19 vaccine effectiveness is immune escape by SARS‐CoV‐2 variants. To overcome this, an Omicron‐specific messenger RNA (mRNA) vaccine was designed. The extracellular domain of the spike of the Omicron variant was fused with a modified GCN4 trimerization domain with low immunogenicity (TSomi). After immunization with TSomi mRNA in hamsters, animals were challenged with SARS‐CoV‐2 virus. The raised nonneutralizing antibodies or cytokine secretion responses can recognize both Wuhan S and Omicron S. However, the raised antibodies neutralized SARS‐CoV‐2 Omicron virus infection but failed to generate Wuhan virus neutralizing antibodies. Surprisingly, TSomi mRNA immunization protected animals from Wuhan virus challenge. These data indicated that non‐neutralizing antibodies or cellular immunity may play a more important role in vaccine‐induced protection than previously believed. Next‐generation COVID‐19 vaccines using the Omicron S antigen may provide sufficient protection against ancestral or current SARS‐CoV‐2 variants.

## 1 INTRODUCTION

Since the outbreak of COVID‐19 began, more than 575 million people worldwide have been infected with SARS‐CoV‐2, and more than 6.4 million people have died as of July 2022.^1^ Many vaccines are available for mass immunization in humans, but the continued emergence of variants of concern (VOCs) is a major challenge for public health. Divergences from the ancestral Wuhan sequence emerged with the B.1.1.7 (Alpha), B.1.351 (Beta), P.1 (Gamma), B.1.1.298 (Delta), and B.1.1.529 (Omicron) variants, and the recent emergence of Omicron raises concerns about whether current vaccines offer cross‐protection against variants. Many mutations in the receptor‐binding domain (RBD) of the spike protein have been identified in the Omicron variant. The Omicron variant has been found to have increased transmissibility and immune evasion after natural infection and vaccination.^2^ Convalescent serum from ancestral, alpha, beta, gamma, and delta strain infections reduced neutralizing antibody titers against the Omicron strain. The neutralization was reduced by 18.4‐fold for serum from alpha, 22.5‐fold for serum from beta, 12.3‐fold for serum from gamma and 25.9‐fold for serum from delta. After the third dose of vaccination with ADZ1222 or BNT162b2, the neutralization titers to Omicron were reduced by 12.7‐fold and 14.2‐fold compared to the ancestral strain, respectively. The neutralization titers to Omicron were reduced 3.6‐fold compared with those to Delta.^3^ At 20–25 weeks after the 2nd‐dose vaccination with AZD1222, BNT162b2, and messenger RNA (mRNA)‐1273, protective efficacy against symptomatic disease caused by the Omicron variant was reduced to 14.9% or less.^4^ These data indicate a substantially reduced neutralizing antibody response to the Omicron variant compared with the ancestral strain of SARS‐CoV‐2 in vaccinated persons. Based on these observations, the current vaccines may be less effective against Omicron variant infection.

Because of the reduced neutralizing activity of current COVID‐19 vaccines, the development of new vaccines to neutralize submicron SARS‐CoV‐2 is critical. Recently, Omicron mRNA vaccines have been developed by different scientists and organizations. The Omicron‐specific mRNA vaccine can induce protective immunity against Omicron virus challenge but fails to induce neutralizing antibodies against other VOCs. Therefore, it will be interesting to investigate whether the Omicron mRNA vaccine candidate can induce protective immunity as an alternative to humoral immunity against other VOCs. This study aims to understand how Omicron mRNA vaccine candidates influence vaccine effectiveness against VOCs. The results will lead to rational vaccine design against SARS‐CoV‐2 infections.

## 2 MATERIALS AND METHODS

### 2.1 Virus titration

SARS‐CoV‐2 variants hCoV‐19/Taiwan/4/2020, classified as the same lineage as the Wuhan‐Hu‐1 reference strain, and hCoV‐19/Taiwan/16804/2021 (omicron) were obtained from the Centers for Disease Control in Taiwan. The virus was cultured and amplified in Vero cells (ATCC CCL‐81) grown in M199 medium (Gibco) containing 2 μg/ml TPCK‐trypsin (Sigma) at 37°C.^5^

### 2.2 mRNA in vitro transcription

The DNA template for mRNA in vitro transcription was linearized pT7TSomi plasmid with restriction enzyme, BamH I and Xbal I (Fermentas). After DNA linearization, the DNA template was resolved in H_2_O to 1 μg/μL and 1 μg for a 40 μL transcription reaction. The N1‐mythel‐pseudoUTP and CleanCap AG 3'Ome (Trilink) were used in this reaction.^6^

### 2.3 mRNA‐LNP preparation

The mRNA‐LNP formulations were prepared using a modified procedure of a previously described method.^7^ Briefly, lipids were dissolved in ethanol at molar ratios of 50:10:38.5:1.5 (SM‐102:DSPC:cholesterol:DMG‐PEG). The lipid mixture was combined with an acidification buffer of 25 mM sodium acetate (pH 5.0) containing mRNA at a volume ratio of 3:1 (water:ethanol) using a microfluidic mixer (Precision Nanosystems).

### 2.4 Animal immunization

Golden Syrian hamsters were obtained from the National Laboratory Animal Breeding and Research Center. K18‐hACE2 transgenic mice were obtained from The Jackson Laboratory. Mice and hamsters were used between 6 and 10 weeks of age. All animals were kept at the Animal Center of the National Health Research Institutes (NHRI) and maintained in accordance with institutional animal care protocols. The animal use protocols were approved by the Institutional Animal Care and Use Committee (IACUC) of the NHRI (Protocol No: NHRI‐IACUC‐110053‐A).

### 2.5 Trimeric spike protein preparation of Omicron

A DNA fragment encoding the Wuhan or Omicron strain S protein was designed to contain a nonfunctional furin cleavage site (R682G, R683S, R685S) and two stabilizing prolines (K986P, V987P) in the hinge loop and a trimeriation domain in the C‐terminal (S‐trimer). The S‐trimer was transiently expressed by Expi CHO cells in serum‐free medium and purified through a Ni2^+^‐NTA agarose column (GE) as described in our previous report.^8^

### 2.6 Neutralization of SARS‐CoV‐2 infection

The sera were diluted to an initial dilution of 1/20 with serum‐free M199 medium, added to a well containing 200 TCID_50_ of SARS‐CoV‐2 in 0.2 ml in Vero cells, and then incubated at 37°C for 2 h. Subsequently, the virus‐serum mixture was inoculated onto Vero cell monolayers and incubated at 37°C. Neutralization titers below the starting dilution of 1:20 were assigned a value of 10 for calculation purposes.^9^, ^10^

### 2.7 ELISpot assay

The T‐cell epitope peptide screening method has been described in a previous report.^5^ In this study, we investigated T‐cell epitope peptides in a C57BL/6 mouse model. C57BL/6 mice were injected intramuscularly twice at a 2‐week interval with TSomi mRNA/LNP (2 μg/mouse). Splenocytes (2.5 × 10^5^ cells) were mixed with 10 μg/ml indicated synthetic peptides derived from Wuhan strain spike protein, and spots were developed using a 3‐amine‐9‐ethyl carbazole (AEC, Sigma, cat# 45‐AEC101‐1KT) solution. The spots were scanned and analyzed by an ELISpot reader (Cellular Technology Ltd.). The synthetic peptides used in the ELSIPOT assay are listed in Supporting Information: Table 1.

### 2.8 Animal challenge

Syrian hamsters (*n* = 8 per group) were immunized with 10 μg of TSomi mRNA vaccine at Days 0 and 14 via i.m. injection. Four weeks after the last vaccination, the hamsters were challenged intranasally with 10^5^ TCID_50_ SARS‐CoV‐2 (hCoV‐19/Taiwan/4/2020) in a 50 μL volume under isoflurane anesthesia. Their body weights (*n* = 4 per group) were recorded every day for 6 days after the challenge. Four hamsters in each group were killed at Day 3 after challenge for viral load quantification.

### 2.9 H&E staining and pathologic score

Lung tissue samples of infected animals were fixed in formalin, dehydrated, and then embedded in paraffin wax by a routine method performed by the core pathology facility at the National Health Research Institutes. To quantify the severity of lung tissue damage, the pathological score was scored as described in a previous study.^11^

### 2.10 Statistical analysis

Statistical data were analyzed by GraphPad Prism software. The statistical significance was determined by the two‐tailed Mann–Whitney test, multiple *t*‐test, two‐way ANOVA and log‐rank (Mantel‒Cox) test.

## 3 RESULTS AND DISCUSSION

**Figure 1:**
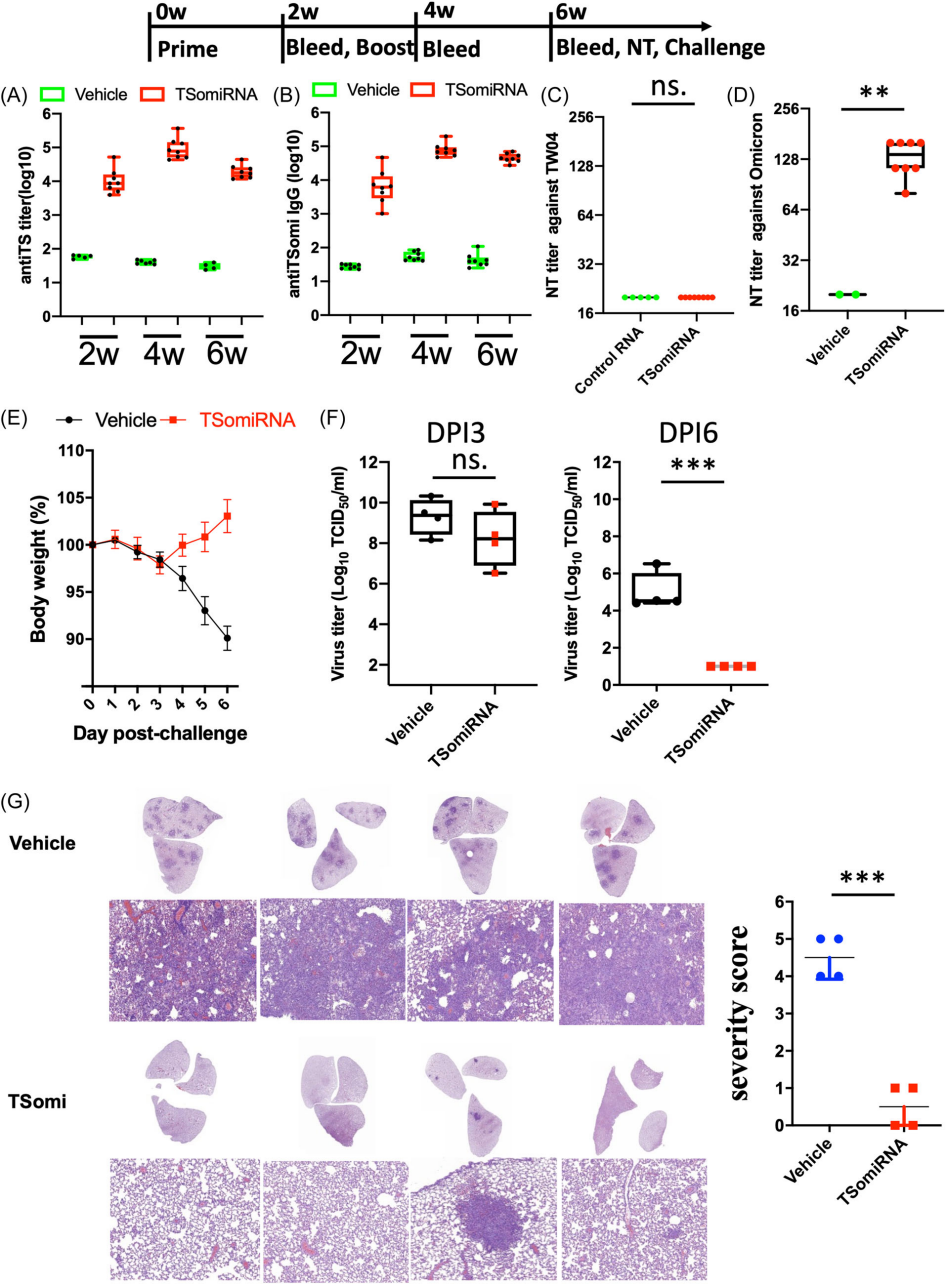
SARS‐CoV‐2 TSomi RNA vaccine immunization induced a protective effect against Wuhan variant challenge in a hamster model. (A) Hamsters were immunized with 10 μg of TSomi mRNA, and serum samples were collected at Weeks 2, 4, and 6 after the first immunization. Total anti‐spike protein IgG titers were determined by (A) SARS‐CoV‐2 Wuhan TS protein or (B) SARS‐CoV‐2 Omicron TS protein antigen‐coating ELISA. 6‐week immunized hamster sera were used to determine neutralizing antibody titers against (C) the Wuhan SARS‐CoV‐2 or (D) the SARS‐CoV‐2 Omicron variant. (E) The TSomi mRNA‐vaccinated hamsters were challenged with Wuhan SARS‐CoV‐2 at Week 6 after immunization. The figure presents the percentage body weight change after virus challenge. (F) Viral titers in the lungs of infected hamsters at DPI 3 and DPI 6 were determined by TCID50 assay. (G) Histopathology of lungs from infected hamsters at 6 DPI was performed by hematoxylin and eosin staining. Scale bar for the low‐magnification image: 1 mm, scale bar for the high‐magnification image: 500 μm. Pathological severity scores of infected hamsters. The *p* value was calculated by *t*‐test. **p < 0.01 and ***p < 0. mRNA, messenger RNA; SARS‐CoV‐2, Severe acute respiratory syndrome coronavirus 2.

We designed a perfusion form of the spike protein of the Omicron variant with a low‐immunogenicity trimeric domain IZN4 derived from GCN4 (TSomi) in the C‐terminal to replace the transmembrane and intracellular domains.^8^, ^12^ The TSomi coding sequence was human‐codon optimized and cloned into pT7ts with a 120‐nt poly‐A tail to generate TSomi mRNA using in vitro transcription. The TSomi mRNA was formulated with lipid nanoparticles (LNPs) for immunization in hamsters. We found that the anti‐Wuhan S antibody titer reached the highest value (log5 ± 0.11) in Week 4 (Figure 1A) and that the anti‐Omicron S antibody titer reached the highest value (log4.6 ± 0.05) in Weeks 4–6 (Figure 1B). In contrast, the sera obtained from TSomi mRNA vaccination achieved little or no neutralization against Wuhan virus (Figure 1C) but did neutralize Omicron virus infection (Figure 1D). These data are consistent with previous reports that sera obtained from Omicron mRNA vaccination induced low cross‐neutralization antibody titers against previous VOCs. Because Omicron variant infection induced mild disease in human^13^ and hamster animal models,^14^ it is interesting to investigate whether TSomi mRNA can induce protective immunity against Wuhan strain virus challenge. Hamsters were immunized twice at 2‐week intervals and challenged with virus at Week 6. The body weight and viral load in the lung were measured. We found that the TSomi mRNA‐vaccinated hamsters did not show decreased body weight compared with the PBS group (+3.05% ± 1.76% vs. −9.89% ± 1.28% on Day 6) (Figure 1E). The viral load in the lung was mildly reduced at Day 3 (log 8.22 vs. log 9.31) and dramatically reduced at Day 6 (log 5.0 vs. log 1.0) (Figure 1F). The pathogenesis in the lung was analyzed at Day 6 and showed severe inflammation in the vehicle immunization group but very low inflammation in the TSomi mRNA vaccination group (Figure 1G). To investigate the long‐term humoral response, Week 20 sera were collected from TS mRNA vaccine‐immunized hamsters with a high neutralization titer against the Wuhan strain (Supporting Information: Figure S1A) but not against the micron‐sized strain (Supporting Information: Figure S1B). In addition, TSomi vaccine boosting induced a high neutralization titer against the omicron variant after 2 weeks (Supporting Information: Figure S1C). The recombinant TS omicron protein bound to ACE2 (KD = 3.7 ± 0.25 × 10^−10^ M) as well as the TS wuhan protein (KD = 3.3 ± 0.18 × 10^−10^ M) (Supporting Information: Figure S2). These data indicated that the TSomi mRNA vaccine can induce protective immunity against Wuhan virus challenge and even raises very low levels of neutralizing antibodies against Wuhan virus.

**Figure 2:**
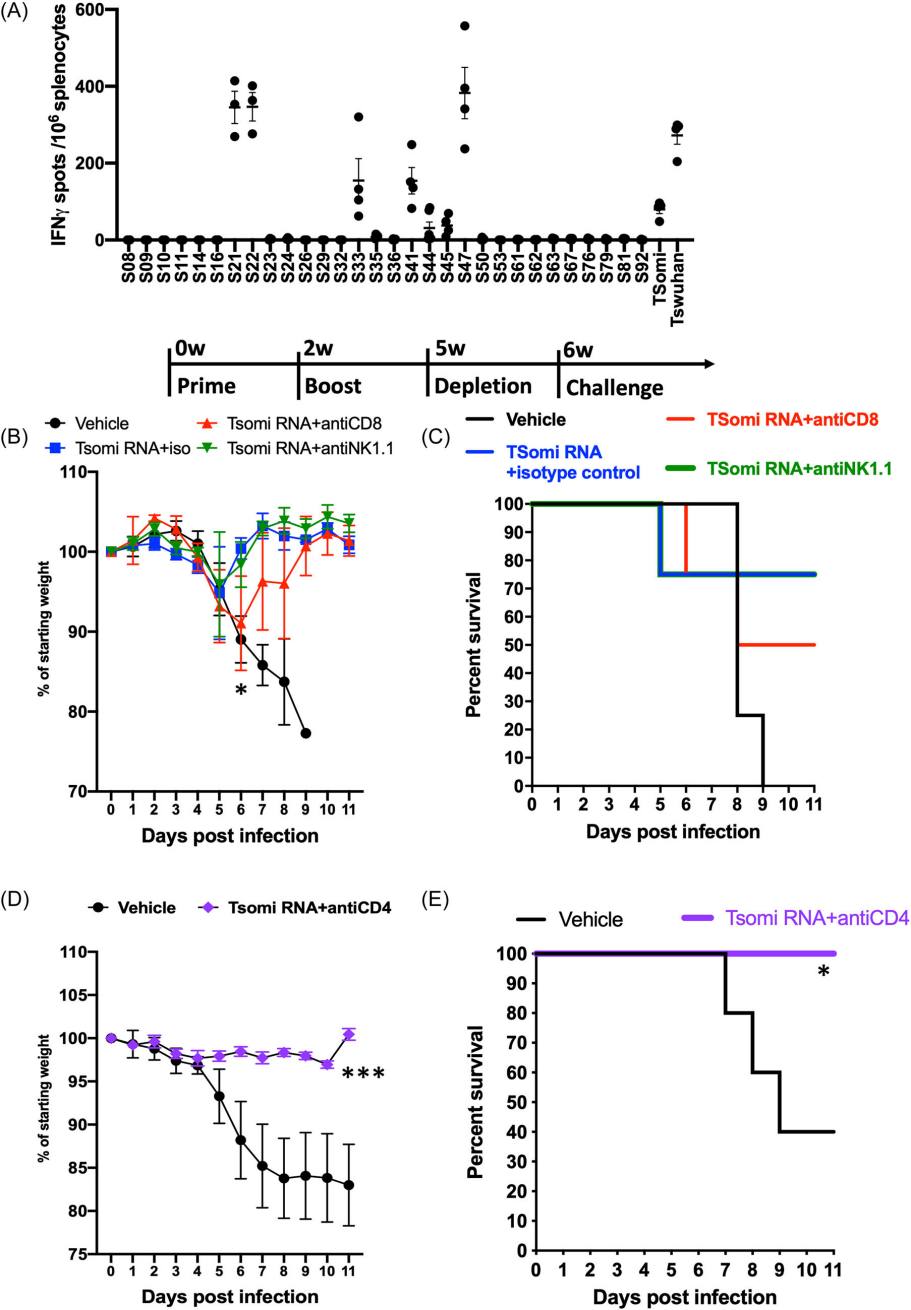
TSomi mRNA vaccination induced protective immunity against Wuhan SARS‐CoV‐2 challenge through the CD8^+^ T‐cell response. C57BL/6 mice were immunized with two doses of 2 μg of TSomi mRNA vaccine at two‐week intervals. (A) At the 4th week after immunization, splenocytes were pulsed with the indicated peptide pool derived from the ancestral Wuhan strain spike protein to induce IFN‐γ production. (B) TSomi mRNA vaccine‐immunized K18‐hACE2 transgenic mice were injected with 200 μg of isotype control IgG2a, anti‐CD8 antibodies (clone 53‐6.7) or anti‐NK1.1 (clone PK136) 7 and 3 days before virus challenge. The K18‐hACE2 transgenic mice were infected with 10^3^ SARS‐CoV‐2 TW04 (Wuhan variant) at 6 weeks after immunization. The percent body weight change in each group was monitored every day. (C) The survival rate of (B) was monitored until 11 DPI (*n* = 4). (D) K18‐hACE2 transgenic mice were immunized with 50 μL of vehicle or 2 μg of TSomi mRNA vaccine at 2‐week intervals. The immunized mice were injected with 200 μg of anti‐CD4 antibodies (clone GK1.5) at 7 and 3 days before virus challenge and 3 and 7 days after virus challenge. The percent body weight change in each group was monitored every day until 11 DPI (*n* = 5). The *p* value was calculated by two‐way ANOVA. (E) The survival rate of (D) was monitored until 11 DPI. The *p* value was calculated by the log‐rank (Mantel‒Cox) test. *p < 0.05 and ***p < 0.001. mRNA, messenger RNA; SARS‐CoV‐2, Severe acute respiratory syndrome coronavirus 2.

To investigate TSomi mRNA vaccine‐induced T‐cell responses, C57BL/6 mice were immunized with 2 μg TSomi mRNA vaccine and then analyzed for specific epitope peptides in the Omicron variant spike protein. The individual peptide‐responsive T cells were determined by IFN‐γ ELISPOT. Figure 2A shows that TSomi mRNA vaccine‐inducing T cells could secrete IFN‐γ upon S21, S22, S33, S41, S44, S45, and S47 peptide restimulation. We found that most of the T‐cell epitopes were conserved between the Wuhan strain and Omicron variant (Table 1). To confirm whether CD8 T cells or NK cells are important in protective immunity, TSomi mRNA vaccine‐immunized K18 mice were depleted of CD8 T cells and NK cells and then challenged with Wuhan virus. We found that two of four CD8 T‐cell depletion K18 mice were moribund with delayed average body weight recovery (Figure 2B). CD8 depletion significantly reduced body weight in the TSomi mRNA vaccination group compared to the isotype control group on DPI6 (*p* < 0.05). In addition, the survival rate of CD8 T‐cell depletion K18 mice decreased to 50% at DPI 8 compared with 75% of isotype control mice and NK depletion K18 mice (Figure 2C). We also subjected CD4‐depleted K18 mice to virus challenge in additional experiments. The body weight of CD4‐depleted TSomi mRNA‐immunized K18 mice remained similar to the initial body weight, in contrast to the decreasing body weight of the vehicle control group (Figure 2D). The 100% survival rate indicated that CD4 depletion did not impair the antiviral effect of the TSomi mRNA vaccine (Figure 2E). In this experiment, we found that the depletion of CD8 T cells partially eliminated the protective effects of TSomi mRNA vaccination.

**Table 1:** Antigenic peptides of omicron variant spike protein

Peptide No.	Amino acid sequence
S21	^261^GAAAYYVGYLQPRTFLLKYNENGTI^285^
S22	^274^TFLLKYNENGTITDAVDCALDPLSE^298^
S33	^417^KIADYNYKLPDDFTGCVIAWNSNNL^441^
S41	^520^PATVCGPKKSTNLVKNKCVNFNFNG^545^
S44	^560^LPFQQFGRDIADTTDAVRDPQTLEI^584^
S45	^573^TDAVRDPQTLEILDITPCSFGGVSV^597^
S47	^599^TPGTNTSNQVAVLYQDVNCTEVPVA^623^

*Note*: Peptides were derived from the SARS‐CoV‐2 Wuhan strain spike protein. The superscript numbers represent the position of the peptide on the Wuhan strain spike protein.

The current COVID‐19 contains the Wuhan strain spike protein as an antigen and does not raise neutralizing antibodies against the Omicron variant.^15^ However, the existing T‐cell immunity may provide protective immunity against the Omicron variant. Boosting with Omicron‐based vaccines may induce neutralizing antibodies against the current pandemic Omicron variant and strongly enhance T‐cell‐mediated protective immunity. In addition, boosting with Omicron‐specific vaccines or infection by Omicron variant may also enhance conserved neutralizing epitopes in the VOCs.^16^ Interestingly, homologous or heterologous boosters using Wuhan‐based vaccines markedly increased neutralizing antibody titers against Omicron sublineages, which may provide sufficient protection against Omicron‐induced severe disease.^17^, ^18^ Except for T‐cell responses, boosting vaccines may increase conserved B‐cell epitope antibody titers through circulating memory B cells against new VOCs.

The mRNA vaccine can elicit robust humoral and cellular immune responses against viral infection. The SARS‐CoV 2 Omicron mRNA vaccine booster may increase the neutralizing antibody titer. In this study, we demonstrated that the TSomi mRNA vaccine‐elicited CD8 T‐cell response plays an important role against SARS‐CoV‐2 infection in hamster and mouse models. In the hamster model, we observed that the TSomi mRNA vaccine could induce an antiviral response with low neutralizing antibody titers. There are three mechanisms of vaccine‐induced protection against SARS‐CoV‐2 infection that include binding antibodies, neutralizing antibodies and CTLs. The neutralizing antibody can block virus entry and reduce virus transmission.^19^ The binding antibody and CTLs are able to clear viruses through cell‐mediated killing.^20^ In the absence of CD8+ T cells, the viral load in the lung cannot be reduced but instead aggravates respiratory distress and/or damage to other organs.^21^ This study may provide some evidence to support that mRNA vaccines could efficiently trigger antigen processing and presentation to elicit T‐cell responses. Taking these data together, the TSomi mRNA vaccine might reduce lung injury by virus infection by eliciting a CD8 T‐cell response. We also observed that NK depletion did not impair TSomi mRNA vaccine‐inducing antiviral efficacy. Therefore, a TSomi mRNA vaccine inducing adaptive immunity might be required for antiviral efficacy. To address future VOCs, boosting vaccines need to include new variants in vaccine components that can boost pre‐existing humoral and cellular immunity for conserved epitopes and generate nonconserved epitopes in new circulating VOCs.

## AUTHOR CONTRIBUTIONS

Kuan‐Yin Shen, Chung‐Hsiang Yang, Ming‐Hsi Huang, and Shih‐Jen Liu designed the experiments, analyzed the data, and prepared the manuscript. Hsin‐Wei Chen and Ching‐Len Liao aided in manuscript review. Chia‐Wei Hsu, Fang‐Feng Chiu, Kuan‐Yin Shen, Chung‐Hsiang Yang, Hui‐Min Ho, and Chiung‐Yi Huang performed the experiments. Chiung‐Tong Chen, Guann‐Yi Yu, Hsin‐Wei Chen, and Hung‐Chun Liao developed reagents. Ching‐Len Liao, Hsin‐Wei Chen, Ming‐Hsi Huang, and Shih‐Jen Liu provided supervision and waived the final manuscript preparation. Kuan‐Yin Shen, Ming‐Hsi Huang, and Shih‐Jen Liu wrote the paper.

## CONFLICT OF INTEREST

The authors declare no conflict of interest.

## Supporting information

Supplementary information.

## Data Availability

Data available on request from the authors.

## References

[1] Center for Systems Science and Engineering (CSSE) at Johns Hopkins University . COVID‐19 Dashboard . 2022. Accessed September 02, 2020. https://coronavirus.jhu.edu/map.html

[2] Fan Y , Li X , Zhang L , Wan S , Zhang L , Zhou F . SARS‐CoV‐2 Omicron variant: recent progress and future perspectives. Signal Transduct Target Ther. 2022;7(1):141. 35484110 10.1038/s41392-022-00997-x PMC9047469

[3] Dejnirattisai W , Huo J , Zhou D , et al. SARS‐CoV‐2 Omicron‐B.1.1.529 leads to widespread escape from neutralizing antibody responses. Cell. 2022;185(3):467‐484. 35081335 10.1016/j.cell.2021.12.046 PMC8723827

[4] Andrews N , Stowe J , Kirsebom F , et al. Covid‐19 vaccine effectiveness against the Omicron (B.1.1.529) variant. N Engl J Med. 2022;386(16):1532‐1546. 35249272 10.1056/NEJMoa2119451 PMC8908811

[5] Tzeng TT , Chai KM , Shen KY , et al. A DNA vaccine candidate delivered by an electroacupuncture machine provides protective immunity against SARS‐CoV‐2 infection. NPJ Vaccines. 2022;7(1):60. 35662254 10.1038/s41541-022-00482-0 PMC9166770

[6] Henderson JM , Ujita A , Hill E , et al. Cap 1 messenger RNA synthesis with co‐transcriptional cleancap((R)) analog by in vitro transcription. Curr Protoc. 2021;1(2):e39. 33524237 10.1002/cpz1.39

[7] Hassett KJ , Benenato KE , Jacquinet E , et al. Optimization of lipid nanoparticles for intramuscular administration of mRNA vaccines. Mol Ther Nucleic Acids. 2019;15:1‐11. 30785039 10.1016/j.omtn.2019.01.013 PMC6383180

[8] Liao HC , Wu WL , Chiang CY , et al. Low‐dose SARS‐CoV‐2 S‐trimer with an emulsion adjuvant induced Th1‐biased protective immunity. Int J Mol Sci. 2022;23(9):4902. 35563292 10.3390/ijms23094902 PMC9101745

[9] Ramakrishnan MA . Determination of 50% endpoint titer using a simple formula. World J Virol. 2016;5(2):85‐86. 27175354 10.5501/wjv.v5.i2.85 PMC4861875

[10] Chai KM , Tzeng TT , Shen KY , et al. DNA vaccination induced protective immunity against SARS CoV‐2 infection in hamsterss. PLoS Neglected Trop Dis. 2021;15(5):e0009374. 10.1371/journal.pntd.0009374 PMC8158926 34043618

[11] Imai M , Iwatsuki‐Horimoto K , Hatta M , et al. Syrian hamsters as a small animal model for SARS‐CoV‐2 infection and countermeasure development. Proc Natl Acad Sci. 2020;117(28):16587‐16595. 32571934 10.1073/pnas.2009799117 PMC7368255

[12] Sliepen K , van Montfort T , Melchers M , Isik G , Sanders RW . Immunosilencing a highly immunogenic protein trimerization domain. J Biol Chem. 2015;290(12):7436‐7442. 25635058 10.1074/jbc.M114.620534 PMC4367253

[13] Chen LL , Chua GT , Lu L , et al. Omicron variant susceptibility to neutralizing antibodies induced in children by natural SARS‐CoV‐2 infection or COVID‐19 vaccine. Emerg Microbes Infect. 2022;11(1):543‐547. 35084295 10.1080/22221751.2022.2035195 PMC8843159

[14] Halfmann PJ , Iida S , Iwatsuki‐Horimoto K , et al. SARS‐CoV‐2 Omicron virus causes attenuated disease in mice and hamsters. Nature. 2022;603(7902):687‐692. 35062015 10.1038/s41586-022-04441-6 PMC8942849

[15] Ai J , Zhang H , Zhang Y , et al. Omicron variant showed lower neutralizing sensitivity than other SARS‐CoV‐2 variants to immune sera elicited by vaccines after boost. Emerg Microbes Infect. 2022;11(1):337‐343. 34935594 10.1080/22221751.2021.2022440 PMC8788341

[16] Quandt J , Muik A , Salisch N , et al. Omicron BA.1 breakthrough infection drives cross‐variant neutralization and memory B cell formation against conserved epitopes. Sci Immunol. 2022;7(75):eabq2427. 35653438 10.1126/sciimmunol.abq2427 PMC9162083

[17] Kaku CI , Champney ER , Normark J , et al. Broad anti‐SARS‐CoV‐2 antibody immunity induced by heterologous ChAdOx1/mRNA‐1273 vaccination. Science. 2022;375(6584):1041‐1047. 35143256 10.1126/science.abn2688 PMC8939765

[18] Bowen JE , Addetia A , Dang HV , et al. Omicron spike function and neutralizing activity elicited by a comprehensive panel of vaccines. Science. 2022;377(6608):890‐894. 35857529 10.1126/science.abq0203 PMC9348749

[19] Bowman KA , Stein D , Shin S , et al. Hybrid immunity shifts the Fc‐effector quality of SARS‐CoV‐2 mRNA vaccine‐induced immunity. mBio. 2022;13:e0164722. 36000735 10.1128/mbio.01647-22 PMC9600672

[20] Britto C , Alter G . The next frontier in vaccine design: blending immune correlates of protection into rational vaccine design. Curr Opin Immunol. 2022;78:102234. 35973352 10.1016/j.coi.2022.102234 PMC9612370

[21] de Candia P , Prattichizzo F , Garavelli S , Matarese G . T cells: warriors of SARS‐CoV‐2 infection. Trends Immunol. 2021;42(1):18‐30. 33277181 10.1016/j.it.2020.11.002 PMC7664351

