# Supplementary material for: Omicron‐specific mRNA vaccine induced cross‐protective immunity against ancestral SARS‐CoV‐2 infection with low neutralizing antibodies
Source: J Med Virol. 2022 Dec 10;95(1):e28370. doi: 10.1002/jmv.28370 (PMC9877661; doi:10.1002/jmv.28370)
Supplement: Supplementary file 1 — Supplementary information. [file JMV-95-0-s001.pdf]

Supplementary table 1.

Peptide no.	Amino acid sequence
S08	^{92}FASTEKSNIIRGWIFGTTLDSKTQS^{116},
S09	^{105}IFGTTLDSKTQSLLIVNNATNVVIK^{129},
S10	^{118}LIVNNATNVVIKVCEFQFCNDPFLG^{142},
S11	^{131}CEFQFCNDPFLGVYYHKNNKSWMES^{155},
S14	^{172}SQPFLMDLEGKQGNFKNLREFVFK^{195},
S16	^{198}DGYFKIYSKHTPINLVRDLPQGFSA^{222},
S21	^{261}GAAAYYVGYLQPRTFLLKYNENGTI^{285},
S22	^{274}TFLLKYNENGTITDAVDCALDPLSE^{298},
S23	^{287}DAVDCALDPLSETKCTLKSFTVEKG^{311},
S24	^{300}KCTLKSFTVEKGIYQTSNFRVQPTE^{324},
S26	^{326}IVRFPNITNLCPFGEVFNATRFASV^{350},
S29	^{365}YSVLYNSASFSTFKCYGVSPTKLND^{389},
S32	^{404}GDEVRQIAPGQTGKIADYNYKLPDD^{428},
S33	^{417}KIADYNYKLPDDFTGCVIAWNSNNL^{441},
S35	^{443}SKVGGNYNYLYRLFRKSNLKPFERD^{467},
S36	^{456}FRKSNLKPFERDISTEIYQAGSTP^{479},
S41	^{520}PATVCGPKKSTNLVKNKCVNFNFNG^{545},
S44	^{560}LPFQQFGRDIADTTDAVRDPQTLEI^{584},
S45	^{573}TDAVRDPQTLEILDITPCSFGGVSV^{597},
S47	^{599}TPGTNTSNQVAVLYQDVNCTEVPVA^{623},
S50	^{638}TGSNVFQTRAGCLIGAEHVNNSYE^{662},
S53	^{678}TNSPRRARSVASQSIIAYTMSLGA^{701},
S61	^{781}VFAQVKQIYKTPPIKDFGGFNFSQI^{805},
S62	^{794}IKDFGGFNFSQILPDPSKPSKRSFI^{818},
S63	^{807}PDPSKPSKRSFIEDLLFNKVTLADA^{831},
S67	^{859}TVLPPLLTDEMIAQYTSALLAGTIT^{883},
S76	^{975}SVLNDILSRLDKVEAEVQIDRLITGR^{1000},
S79	^{1015}AAEIRASANLAATKMSECVLGQSKR^{1039},
S81	^{1041}DFCGKGYHLMSFPQSAPHGVVFLHV^{1065},
S92	^{1184}DRLNEVAKNLNESLIDLQELGKYEQ^{1208},

1. Peptides were derived from SARS-CoV2 Wuhan variant spike protein.
2. The superscript numbers represent the position of peptide on wuhan variant spike protein

Figure S1

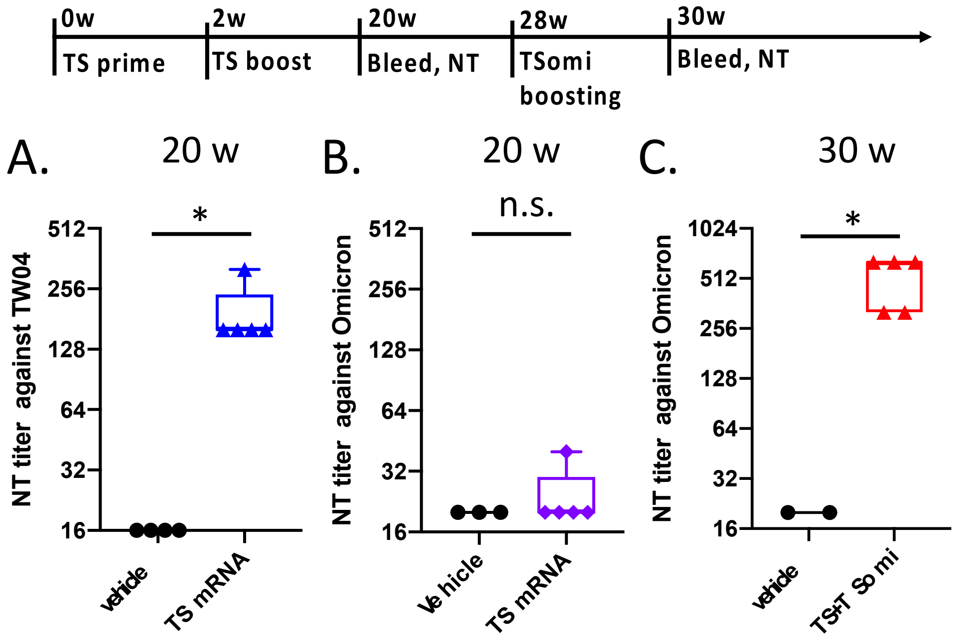

Figure S1. The long-term neutralizing antibody titer evaluation of TS mRNA vaccine in hamster model. Hamsters were immunized with 10 µg TS mRNA vaccine at two-week intervals. The week 20 sera were against (A) TW04 (wuhan strain) or (B) omicron strain to evaluate neutralizing antibody titer. (C) The TS mRNA-immunized hamsters were boosting with 10 µg TSomi vaccine at week 28. After boosting, the week 30 sera were evaluated neutralizing antibody titer against omicron strain. (n=5, * represent $p<0.05$)

Figure S2

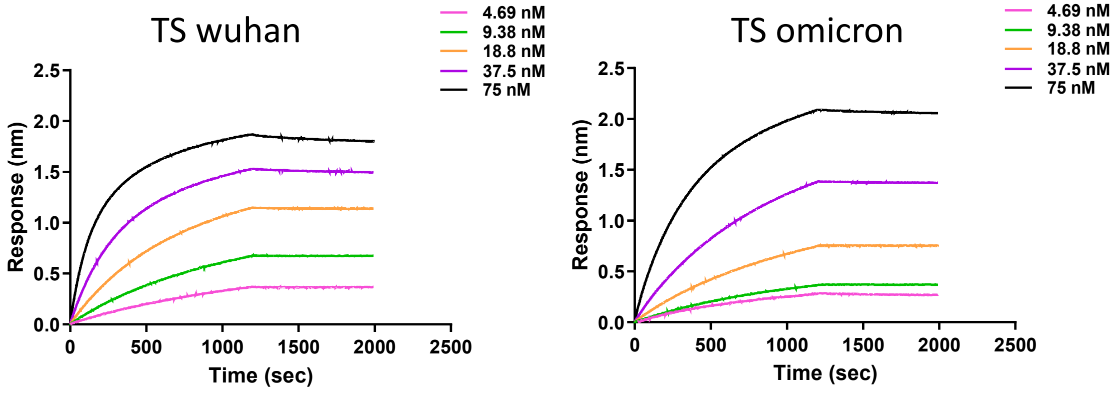

***KD* = 3.3 ± 0.18 x 10^{-10} M**
***Ka* = 6.3 ± 0.02 x 10^{4} Ms^{-1}**
***Kd* = 2.1 ± 0.11 x 10^{-5} s^{-1}**

***KD* = 3.7 ± 0.25 x 10^{-10} M**
***Ka* = 3.3 ± 0.006 x 10^{4} Ms^{-1}**
***Kd* = 1.2 ± 0.08 x 10^{-5} s^{-1}**

Figure S2. The binding affinity of ACE2 between recombinant TS protein or TS omicron protein. The recombinant TS protein and TS omicron protein were expressed by CHO cell system and then purified by Ni-affinity column into tris buffer. The binding affinity of human ACE2 to TS protein or TS omicron protein was measured by ForteBio BioLayer Interferometry (BLI). The data representative of at least three independent experiments.
